# Infants’ Hemodynamic Modulation in the Temporal Region

**DOI:** 10.3389/fnhum.2022.821539

**Published:** 2022-03-10

**Authors:** Yuki Tsuji, So Kanazawa, Masami K. Yamaguchi

**Affiliations:** ^1^Research and Development Initiative, Chuo University, Tokyo, Japan; ^2^Department of Psychology, Japan Women’s University, Tokyo, Japan; ^3^Department of Psychology, Chuo University, Tokyo, Japan

**Keywords:** fNIRS, infant, right occipitotemporal, modulation, Peekaboo

## Abstract

This study examined whether 8-month-old infants’ hemodynamic responses in the temporal region were modulated by repeated presentation of “Peekaboo” by using functional near-infrared spectroscopy (fNIRS). Previous studies have shown that infants’ temporal region responds to faces (e.g., Otsuka et al., 2007). A recent electroencephalography study showed that the neural activity of infants was modulated by repeated presentation of “Peekaboo.” Some fNIRS studies also revealed that the movie of “Peekaboo” activated the hemodynamic response of the temporal region in infancy. However, no studies have shown the hemodynamic modulation of the temporal region according to the repeated presentation of “Peekaboo” in infants. In order to examine whether the hemodynamic responses of the temporal region were modulated by repeated presentation of “Peekaboo,” we compared the activity of the temporal region between the early and late trials. We set long and short delays before face-presentation. The results showed that the concentration of oxy-hemoglobin in the right occipitotemporal region (Ch 21) in both conditions increased after the presentation of “Peekaboo” relative to the baseline. Moreover, in the long delay condition, the hemodynamic modulation of the right occipitotemporal region was induced according to the repeated presentation of “Peekaboo” in infants.

## Introduction

Infants, as well as adults, have brain regions that show specific responses to facial images. Lloyd-Fox et al. (2009) measured 5-month-old infants’ hemodynamic responses to images of faces and objects in the temporal regions using functional near-infrared spectroscopy (fNIRS), and found that the concentration of oxy-hemoglobin (oxy-Hb) increased only for facial stimuli. Honda et al. (2009) measured the hemodynamic responses of 7-and 8-month-old infants to images of canonical and scrambled faces in the temporal regions using fNIRS, and demonstrated that the concentration of total-Hb in the right temporal region significantly increased for the canonical face. These studies indicate that the temporal region responds to images of faces. Moreover, Otsuka et al. (2007) measured the hemodynamic responses of 5–8-month-old infants to images of upright and inverted faces in the temporal regions using fNIRS, and showed that the concentration of oxy-Hb and total-Hb in the right temporal region significantly increased for upright faces compared to inverted faces. This study indicated that temporal regions respond to a typical view of the face. Kobayashi et al. (2012) measured the hemodynamic responses of 7− and 8-month-old infants to upright faces and inverted Arcimboldo images using fNIRS. Arcimboldo images are known to induce face perception despite being composed of a variety of non-facial objects (fruits and vegetables). They showed that the concentration of oxy-Hb increased in the left temporal area during the presentation of upright Arcimboldo images, indicating that temporal regions respond even to face-like objects. Therefore, infants’ temporal region was activated not only when exposed to facial photographs, but also non-real faces such as face-like objects. In our study, we examined whether the hemodynamic responses of the temporal region were modulated by the repeated presentation of the face, something which has not been done in previous studies. Generally, the repeated presentation of an identical face attenuates the hemodynamic response (Kobayashi et al., 2014). However, Peekaboo, in which one’s face is first hidden and then shown, is very attractive for infants. Thus, the repeated presentation of “Peekaboo” may increase the hemodynamic response.

A recent electroencephalography (EEG) study by Mento and Valenza (2016) reported that repeated presentation of the face modulated event-related potential (ERP) activity in infants and adults. They measured EEG during an experimental task simulating a “Peekaboo” as the repeated presentation of the face. They conducted two experimental conditions: standard and delayed. In both conditions, images of one of the four female models was randomly chosen for each trial. In the standard condition, the image of a female covering her face with hands was presented for 600 ms with three consecutive beeping sounds (3 × 200 ms) followed by a smiling face of a female for 500 ms with a beeping sound (200 ms). In the delayed condition, the stimuli were the same as those in the standard condition, except for the image of a female covering her face with hands that lasted 2,100 ms with the simultaneous presentation of three consecutive beeping sounds (3 × 200 ms). The standard condition was presented 8 times in the learning phase, and in the test phase, the standard condition was presented 54 times, and the delayed condition 16 times (70 total trials). Overall, four blocks were presented. They measured ERP activity during the presentation of the image of a female covering her face with hands in the delayed condition and found the Contingent Negative Variation (CNV) in infants and adults. In adults, an EEG study indicated that CNV was related to the subject’s anticipation of an upcoming event (Walter et al., 1964; Vogel and Luck, 2000). Interestingly, the CNV amplitude in the posterior region from trials in the late experimental period was greater than that in the early experimental period in the posterior region. This result indicated that infants’ CNV amplitude in the posterior region was modulated by repeated presentation of “Peekaboo.” However, whether the activity of temporal regions was modulated by repeated presentation of “Peekaboo” has not been clarified.

The movie of “Peekaboo” has been used to measure infants’ hemodynamic response by using fNIRS (Lloyd-Fox et al., 2009, 2013, 2014, 2017, 2018). “Peekaboo” and “Incy Wincy Spider” were presented during the test period, and still images of different types of transport were displayed randomly for a pseudorandom duration (1–3 s) during the baseline period (Lloyd-Fox et al., 2009). The researchers compared infants’ brain activities between the test and baseline periods, and found that the concentration of oxy-Hb in the temporal region significantly increased during the test period. The results showed that the hemodynamic responses of the temporal region were activated by the movies of “Peekaboo” and “Incy Wincy Spider.” As mentioned earlier, an EEG study showed that neural activity (CNV) was modulated by repeated presentation of “Peekaboo.” In the present study, we investigated whether infants’ hemodynamic responses in the temporal region are modulated by repeated presentation of “Peekaboo” by using fNIRS.

In our experiment, we designed two “Peekaboo” conditions; short and long delays before face-presentation. The long delay condition simulating a “Peekaboo” was compared to the short delay condition. In the long delay condition, to examine whether the hemodynamic responses were modulated, the hemodynamics of the temporal regions were compared between the early and late trials.

## Materials and Methods

### Participants

The final sample included 16 infants who were 8 months old (10 females, aged 223–269 days, mean age of 247 days). We finalized this sample size based on previous NIRS studies that used a similar experimental task (Kobayashi et al., 2018; Tsurumi et al., 2019). Another eight infants participated but were not included in the final analyses because of an insufficient number of available trials (fewer than six trials for either the short or long delay condition) owing to crying, failure to look at the stimuli or motion artifacts. The minimum number of valid trials was set to six to investigate the change of hemodynamic response by repetition. The infants were recruited through newspaper advertisements. All the infants were full-term at birth and healthy at the time of the experiment. Written informed consent was obtained from the parents of the participants. The study protocol was approved by the Ethical Committee of Chuo University (2020-39). This study was conducted according to the principles and guidelines of the Declaration of Helsinki. Parents gave prior written informed consent for their children’s participation and for the publication of the results in an online open-access publication.

### Stimuli and Design

**FIGURE 1:**
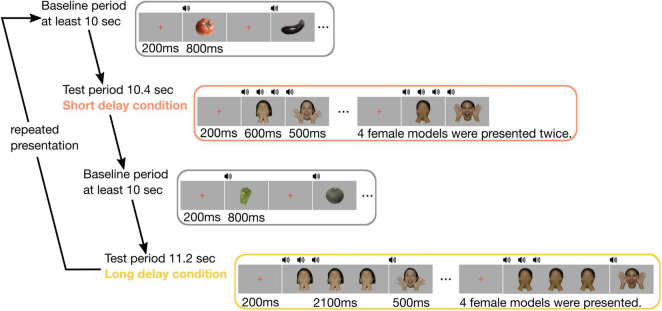
Experimental procedure. In each trial, the baseline period comprised images of five vegetables, and its duration was for at least 10 s. The test period included images of four female faces. In the short delay condition, four female faces were presented twice, and its duration was 10.4 s. In the long delay condition, four female faces were presented, and its duration was 11.2 s.

The sequence of stimuli presentation consisted of two test periods (short and long delay conditions) and a baseline period (Figure 1). In both the conditions, the stimuli for the test period consisted of color photo images of six Japanese females smiling and covering their faces with their hands. We used images from four of the six female models who were randomly chosen for each trial. In the short delay condition, we first presented a red cross for 200 ms. Subsequently, a female covering her face with hands was presented for 600 ms with three consecutive beeping sounds (3 × 200 ms), and a smiling female face was presented for 500 ms with a single beeping sound (200 ms). This sequence was repeatedly presented eight times. The presentation of stimuli in the long delay condition was similar to that in the short delay condition, except for extending the duration of a female covering her face with her hands up to 2,100 ms. This sequence was repeatedly presented four times. The stimuli for the baseline period consisted of color photo images of five vegetables. For the baseline period, the image of each vegetable was presented for 800 ms with a single beeping sound (200 ms), and the 200 ms inter-stimulus interval was filled by the presentation of a red cross. The intertrial interval was controlled by the experimenter, and the duration was at least 10 s. The images of female face stimuli were 13.3 × 17.9 cm and the vegetable stimuli were 14.5 × 16.0 cm in size.

### Apparatus and Procedure

The experimental stimuli were presented on a 32-inch LCD monitor (Display++, Cambridge Research Systems, 1,920 × 1,080 pixel resolution, refresh rate of 120 Hz) using PsychoPy 3.0. The infants sat on their parents’ lap approximately 50 cm away from the screen.

A camera was set up below the display to monitor and record the infants’ behavior while looking at the stimuli. An experimenter could observe the infants’ behavior through a monitor connected to the camera.

### Functional Near-Infrared Spectroscopy Recordings

Throughout the experiment, fNIRS measurements were performed. A multi-channel fNIRS unit operating at 695 and 830 nm wavelengths (ETG-4000 system, Hitachi Medical, Chiba, Japan) was used to measure temporal changes in the concentrations of oxy-Hb, deoxy-Hb, and total-Hb from 24 channels with 0.1-s time resolution. Twelve channels each were assigned for the measurement of the right and the left temporal cortex. The fNIRS probes (Hitachi Medical) included nine optical fibers (3 × 3 arrays) with five emitters and four detectors. The optical fibers were held in place with a soft silicon holder. The distance between the emitters and detectors was set to 2 cm. Each pair of adjacent emitting and detecting fibers is defined as a single measurement channel. We set the probes at the same location of the bilateral temporal cortices, as in our previous studies (Honda et al., 2009; Nakato et al., 2009; Ichikawa et al., 2010, 2019; Kobayashi et al., 2012). The central positions of the probes were T5 and T6, according to the International 10–20 system (Jasper, 1958). This position in adults is greater than the superior temporal sulcus (Homan et al., 1987).

### Data Analysis

We eliminated the trials from the analysis (1) if the infants did not look at the test stimuli for more than 8 s, or (2) if they became fussy. Additionally, (3) trials in which the infants looked back at the face of the experimenter during the preceding baseline period, and (4) trials in which movement artifacts were detected by the analysis of sharp changes in the fNIRS raw time series were isolated from the analysis. The raw oxy-Hb data from individual channels were digitally filtered with 0.02–1 Hz bandpass filter to eliminate longitudinal signal drift and noise from the instrument.

Subsequently, the mean concentration recorded from each channel was calculated for each subject by averaging the data across the trials in a time series (at a temporal resolution of 0.1-s) starting 1 s before the trial onset up to 15 s after trial offset.

We calculated the Z-score for oxy-Hb concentration in the short and long delay conditions for each channel for each subject based on the mean concentrations in the time series. The Z-score (z) was calculated as the difference between the mean concentrations during the baseline (*m*_*baseline*_) and the test period (*m*_*test*_), divided by the standard deviation of the baseline data (*sd*):


$$Z=\left(m_{t\,e\,s\,t}-m_{b\,a\,s\,e\,l\,i\,n\,e}\right)/s\,d$$

The mean concentration value recorded 1 s immediately before each test trial was used as the baseline concentration, similar to the protocol used in a previous study. Although the fNIRS raw data were originally relative values and could not be averaged directly across participants or channels, the normalized data such as the Z-scores could be averaged, regardless of the unit of measurement.

### Statistical Analysis

First, we performed statistical analyses on the mean Z-scores from 8 s after stimulus onset to a stimulus offset for each condition (Ch 1–24). Previous studies of infants have shown that a latency of up to 10 s in oxy-Hb was observed by presenting dynamic facial images (11–17 s: Ichikawa et al., 2010; 10–18 s: Lloyd-Fox et al., 2009). These findings suggest that the peak of neural response to dynamic stimuli was expected to emerge later after stimulus onset. To determine significant activated channels, a two-tailed one-sample *t*-test on a chance level of 0 (baseline) was conducted for the Z-scores for each channel from 8 s after stimulus onset to stimulus offset for each condition.

Second, we compared the Z-scores of the early and late experimental periods for each significantly activated channel. We defined the first three valid trials as the early experimental period, and the last three as the late experimental period. We again calculated the mean Z-scores from 8 s after stimulus onset to stimulus offset in the two experimental periods. To compare the early and late experimental periods for each significantly activated channel, Z-scores were submitted to 2-way repeated measures analysis of variance (ANOVA), with condition (short or long) and time (early or late) as a within-subjects factor.

## Results

We analyzed hemodynamic responses obtained from 16 infants who observed the stimuli in three or more trials in both the short and long delay conditions. The mean numbers of valid and invalid trials are listed in Table 1.

**TABLE 1:** Means and standard deviations of the number of valid and invalid trials for each condition.

	Valid		Invalid	
	Mean	*SD*	Mean	*SD*
Short delay	6.2	0.44	3.4	0.93
Long delay	6.8	1.00	2.7	0.85

### Identification of Activated Channels (*t*-test)

**FIGURE 2:**
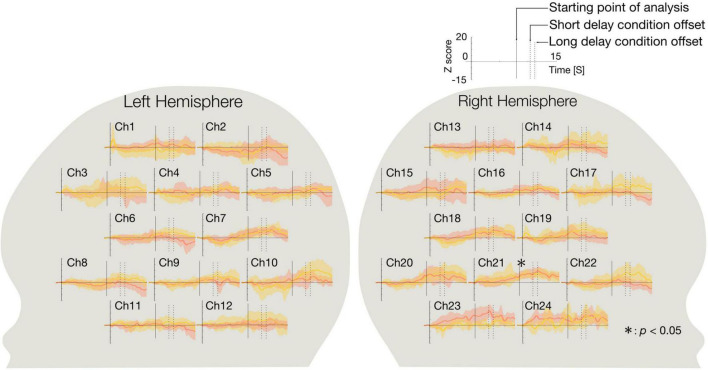
The time course of the average Z-score of oxy-Hb during the short and long delay conditions. The graphs on the left side show the time course of the average Z-score of the concentration of oxy-Hb changes in each channel in the left hemisphere, and those on the right side show the time course of the average Z-score of the concentration of oxy-Hb changes in each channel in the right hemisphere. The red line represents the time course of the average Z-score of the short delay condition and the yellow line represents the time course of the average Z-score of the long delay condition. Shaded regions of red and yellow indicate 95% confidence intervals. On the horizontal axis, 0 represents the onset of the presentation of the stimulus. Intersections of the horizontal axis and solid line represent starting point of analysis (8.0 s), and dotted lines represent the offset of the stimulus of the short delay condition (10.4 s), and the long delay condition (11.2 s). In both conditions, the concentration of oxy-Hb at Ch 21 (right occipitotemporal region) were significantly greater relative to the baseline (*p* < 0.05). Asterisk indicates the significance level of statistical differences: *p < 0.05.

We identified significant activated channels compared to the baseline under each condition using a two-tailed *t*-test. Under each condition, a two-tailed *t*-test for mean Z-scores of the concentration of oxy-Hb from 8 s after stimulus onset to stimulus offset (Ch 1–24) was performed against the baseline. Figure 2 shows the time course of the average change Z-scores in the short and long delay conditions for each channel. At Ch 21 (right occipitotemporal region), the Z-scores in both conditions significantly increased relative to baseline [short delay condition: *t*(15) = 4.4, *p* < 0.05, Cohen’s *d* = 1.1, power (1-β) = 0.63, long delay condition: *t*(15) = 5.1, *p* < 0.05, Cohen’s *d* = 1.3, power (1-β) = 0.81, Bonferroni corrected: *p* = 0.05/48 (2 conditions × 24 channels)]. No other significantly activated channel was observed.

### Conditions Effect on Hemodynamic Response (Z-Scores Were Submitted to 2-Way Repeated Measures Analysis of Variance)

**FIGURE 3:**
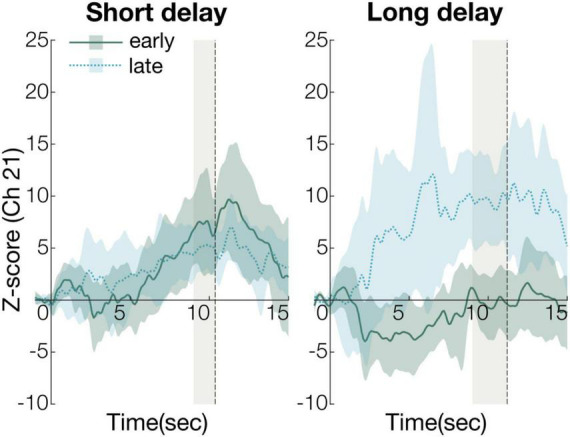
The time course of the average change of Z-scores in the early and late periods (Ch21). The solid green line in the graph represents the time course of the average Z-score in the early period and the dashed blue represents the time course of the average Z-score in the late period. The shaded regions of green and blue indicate 95% confidence intervals. The gray shaded region represents statistical analysis time-window (short delay condition: 8–10.2 s, long delay condition: 8–11.4 s).

To compare the early and late experimental periods on significantly activated channels (Ch21, Figure 3), we defined the first three valid trials as the early experimental period, and the last three as the late experimental period. We calculated Z-scores from 8 s after stimulus onset to stimulus offset (gray shaded regions in Figure 3) for the two experimental periods.

**FIGURE 4:**
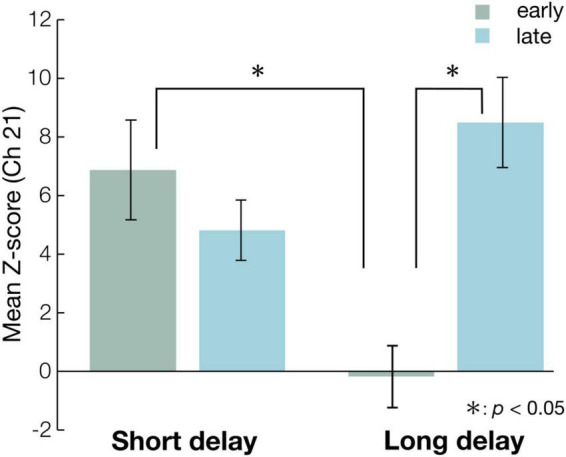
The mean Z-scores of Ch 21 at each condition in the early and the late period. The blue bars represent the mean Z-scores in the statistical analysis time-window (see Figure 3) of each condition in the early period, while the green bars represent the mean Z-scores in statistical analysis time-window (see Figure 3) of each condition in the late period. The error bar represents standard error. Asterisks indicate the significance level of statistical differences: *p < 0.05.

The Z-scores were submitted to 2-way repeated measures analysis of variance (ANOVA), with condition (short or long) and time (early or last) as a within-subject factor. The ANOVA revealed a significant main effect of condition [*F*_(1, 15)_ = 4.8, *p* < 0.05, η*_*p*_*^2^ = 0.24, power (1-β) = 0.54] and interaction of condition and time [*F*_(1, 15)_ = 23, *p* < 0.01, η*_*p*_*^2^ = 0.60, power (1-β) = 0.95]. *Post-hoc* analyses revealed two significant, simple effects. In the early period, the Z-scores of the short and long delay conditions were significantly different [*F*_(1, 30)_ = 13, *p* < 0.01, η*_*p*_*^2^ = 0.30, power (1-β) = 0.69]. This indicates that the Z-score of the short delay condition was larger than that of the long delay condition in the early period (Figure 4). In the late period, the Z-scores of the short and long delay conditions were not significantly different [*F*_(1, 30)_ = 3.6, *p* > 0.05, η*_*p*_*^2^ = 0.11, power (1-β) = 0.51]. The Z-scores in the late and early periods were significantly different [*F*_(1, 28)_ = 21, *p* < 0.01, η*_*p*_*^2^ = 0.42, power (1-β) = 0.97] in the long delay condition. The Z-score of the late period was greater than that of the early period in the long delay condition (Figure 4). In the short delay condition, the Z-scores of the early and late periods were not significantly different [*F*_(1, 28)_ = 1.2, *p* > 0.05, η*_*p*_*^2^ = 0.041, power (1-β) = 0.20].

## Discussion

In this study, we examined whether the repeated presentation of “Peekaboo” modulated infants’ brain activities, and compared 8-month-old infants’ hemodynamic responses in the temporal region between the short and long delay conditions. The short delay condition had a shorter duration before face presentation, while the long delay condition had a longer duration before face presentation. First, we identified significant activated channels compared with the baseline under each condition. We found that the Z-score of the concentration of oxy-Hb in both conditions was significantly increased relative to the baseline at Ch21 (right occipitotemporal region). Specifically, the hemodynamic response of the right occipitotemporal region was activated in both conditions. Second, we defined the first three valid trials as the early experimental period, and the last three as the late experimental period, and compared the Z-scores in the early and late experimental period on Ch21. The Z-scores of the short and long delay conditions were significantly different during the early period. This result showed that the Z-score of the short delay condition was greater than that of the long delay condition in the early period. In contrast, there were no differences between the short and long delay conditions in the late period. Moreover, in the long delay condition, the Z-scores of the early and late periods were significantly different, while in the short delay condition, the Z-scores of the early and late periods were not different. This result demonstrates that the Z-score of the late period was greater than that of the early period only in the long delay condition, suggesting that the right occipitotemporal region (Ch21) activity in the late period was greater than that in the early period in the long delay condition. The repeated presentation of “Peekaboo” modulated infants’ hemodynamic response in the long delay condition.

Even in the short and long delays before face presentation, we found that the right occipitotemporal region was significantly activated to the “Peekaboo.” Previous fNIRS studies have shown that the hemodynamic response of the right temporal region responded to facial stimuli. Recently, Lloyd-Fox et al. (2009, 2013, 2014, 2017, 2018) measured infants’ hemodynamic response to mixed social stimuli, including “Peekaboo,” using fNIRS. They measured infants’ hemodynamic response for face (“Peekaboo”) and hand (“Incy Wincy Spider”) movement combined into social stimulus, and identified activity in bilateral temporal regions. We assume that the activity in bilateral temporal regions may be induced by not only facial stimuli, but also hand movements. In comparison to their experiment, we used only the face (“Peekaboo”) in our experiment. Therefore, we presume that the activation of the right occipitotemporal region was induced by a simple face-presentation of “Peekaboo.”

We found that the hemodynamic responses of the right occipitotemporal region of the short and long delay conditions were significantly different in the early period. This suggests that the short delay condition induced stronger activation than the long delay condition in the early period. We assume that the number of face presentations (total presentation time) enhanced activity in the early period of the short delay condition. In the short delay condition, the number of face presentations in each trial was eight (total 4,000 ms), while in the long delay condition, it was four (total 2,000 ms). A previous functional magnetic resonance imaging (fMRI) study showed a linear correlation between the number of visual stimuli in a trial and the hemodynamic responses [blood oxygenation level-dependent (BOLD) signal] (Dale and Buckner, 1997). They measured the hemodynamic responses evoked by the presentation of one, two, or three identical visual stimuli (full-field visual checkerboard stimulation for 1 s) at short interstimulus intervals (2 s) in a trial using fMRI. The results showed that the total hemodynamic responses increased linearly as the number of stimuli in the trial increased. Many studies have demonstrated a high correlation between fNIRS (oxy-Hb) and fMRI (BOLD) signals (Yamamoto and Kato, 2002; Strangman et al., 2002; Hoge et al., 2005). Therefore, we presume that the difference in the number of face presentations in each trial between the short and long delay conditions would induce a difference in the hemodynamic response between the short and long delay conditions in the early period. However, only in the long delay condition did the hemodynamic response increase from early to late period; this presentation number effect disappeared in the late period, as mentioned below. Specifically, in the late period, we found no difference between the short and long delay conditions.

In the long delay condition, we found that the hemodynamic responses of the right occipitotemporal region in the early and late periods were significantly different, while there was no difference in the short delay condition. This suggests that in the long delay condition, stronger activation was induced in the late period than in the early period. We assume that in the long delay condition, the repeated presentation of “Peekaboo” would induce activation of the right occipitotemporal region in the late period. Mento and Valenza (2016) showed that the amplitude of ERP (CNV) before face presentation was increased by the repeated presentation of “Peekaboo.” The CNV has been considered an ERP associated with anticipation of upcoming events (Walter et al., 1964; Vogel and Luck, 2000). In general, ERP has exceptional temporal resolution and can track rapid temporal modulations in neural activity. In this study, we applied a delay in face presentation such as Mento and Valenza’s “Peekaboo” experiment, and measured hemodynamic responses using fNIRS. The fNIRS has a low temporal resolution and thus it is difficult to demonstrate temporal modulations. However, the fNIRS has a high spatial resolution, and thus could show the hemodynamic response of specific areas such as the right occipitotemporal region. Our fNIRS results indicate modulation of hemodynamic response in the right occipitotemporal cortex. Many fNIRS studies have reported that infants’ temporal regions are activated on various faces. This study also reported face-related hemodynamic response in temporal regions. However, this is the first study to show hemodynamic modulation according to the repeated presentation of the face.

## Conclusion

The present study provided two novel findings concerning 8-month-old infants’ hemodynamic response using fNIRS. First, we found that the infants’ hemodynamic response in the right occipitotemporal region (Ch 21) increased by the presentation of “Peekaboo” relative to the baseline. Second, we compared mean hemodynamic responses at this channel between the first three valid trials (early period) and the last three valid trials (late period) in each condition. In the long delay condition, which has a long duration before the face-presentation, the activation of the right occipitotemporal region was stronger in the late period than in the early period. This finding indicates that only in the long delay condition, infants’ hemodynamic response of the right occipitotemporal region was modulated, according to the repeated presentation of “Peekaboo.” We speculate that this modulation of the hemodynamic response is associated with the infants’ ability of learning regularity and prediction, indicating the attractiveness of “Peekaboo” for babies, although further research is needed to determine this possibility.

## Data Availability Statement

The raw data supporting the conclusions of this article will be made available by the authors, without undue reservation.

## Ethics Statement

The studies involving human participants were reviewed and approved by the Ethical Committee of Chuo University. Written informed consent was obtained from the participants’ legal guardian/next of kin for the publication of any potentially identifiable images or data included in this article.

## Author Contributions

YT, SK, and MY designed the experiments and wrote the manuscript. YT performed the experiments and analyzed the data. All authors contributed to the article and approved the submitted version.

## Conflict of Interest

The authors declare that the research was conducted in the absence of any commercial or financial relationships that could be construed as a potential conflict of interest.

## Publisher’s Note

All claims expressed in this article are solely those of the authors and do not necessarily represent those of their affiliated organizations, or those of the publisher, the editors and the reviewers. Any product that may be evaluated in this article, or claim that may be made by its manufacturer, is not guaranteed or endorsed by the publisher.
